# Physical Training Status Determines Oxidative Stress and Redox Changes in Response to an Acute Aerobic Exercise

**DOI:** 10.1155/2016/3757623

**Published:** 2016-03-15

**Authors:** Farnaz Seifi-skishahr, Arsalan Damirchi, Manoochehr Farjaminezhad, Parvin Babaei

**Affiliations:** ^1^Department of Sport Physiology, Faculty of Physical Education and Sport Sciences, University of Guilan, Rasht, Iran; ^2^Department of Physical Education and Sport Sciences, Faculty of Education and Psychology, Mohaghegh Ardabili University, Ardabil, Iran; ^3^Medicinal Plants Research Center, Ardabil Branch, Islamic Azad University, Ardabil, Iran; ^4^Department of Physiology, Faculty of Medicine, Guilan University of Medical Sciences, Rasht, Iran; ^5^Cellular & Molecular Research Center, Guilan University of Medical Sciences, Rasht, Iran

## Abstract

*Objective*. To assess the influence of different physical training status on exercise-induced oxidative stress and changes in cellular redox state.* Methods*. Thirty male subjects participated in this study and were assigned as well-trained (WT), moderately trained (MT), and untrained (UT) groups. The levels of cortisol, creatine kinase, plasma reduced glutathione to oxidized glutathione (GSH/GSSG), cysteine/cystine (Cys/CySS), and GSH/GSSG ratio in red blood cells (RBCs) were measured immediately and 10 and 30 min after exercise.* Results*. Following the exercise, plasma GSH/GSSG (*p* = 0.001) and Cys/CySS (*p* = 0.005) were significantly reduced in all groups. Reduction in plasma GSH/GSSG ratio in all groups induced a transient shift in redox balance towards a more oxidizing environment without difference between groups (*p* = 0.860), while RBCs GSH/GSSG showed significant reduction (*p* = 0.003) and elevation (*p* = 0.007) in UT and MT groups, respectively. The highest level of RBCs GSH/GSSG ratio was recorded in MT group, and the lowest one was recorded in the WT group.* Conclusion*. Long term regular exercise training with moderate intensity shifts redox balance towards more reducing environment, versus intensive exercise training leads to more oxidizing environment and consequently development of related diseases.

## 1. Introduction

The redox state represents the oxidation/reduction potential within the cell and plays an important role in cells function [1]. The three most important redox systems are nicotinamide adenine dinucleotide phosphate (NADPH/NADP+), thioredoxin (reduced/oxidized TRX), and glutathione (reduced/oxidized glutathione). Glutathione is one of the most important antioxidants, protecting tissues from oxidative damage [2], and helps to maintain homeostasis [3] and redox status [4]. Generally, in all redox systems, the relative amounts of reduced (more negative) and oxidized (more positive) form determine cellular redox state [2] and more reduced redox represents healthy status [5], while more oxidized form predisposes individuals to aging and diseases [6, 7]. Therefore, the ratio of GSH and GSSG is in the core of the redox hypothesis [8, 9] and directly reflects intracellular redox alterations at resting and provocative states [10].

It has been known that exercise of sufficient volume, intensity, and duration can induce oxidative stress [11] and leads to disturbing cellular metabolism and function [12, 13]. In other words, the conditions that favor accelerated production of free radicals cause a permanent shift in redox balance towards a more oxidized environment [6] and lead to disturbing proteins and lipids [14]. On the other hand, the low-molecular-weight thiol/disulfides, such as GSH/GSSG and Cys/CySS, exist under nonequilibrium states in which the kinetics of oxidation and reduction determine the steady-state balance of reduced and oxidized forms [15]. This displacement from equilibrium allows rapid and dynamic regulation, supports redox signaling, and represents a central target of nonradical mechanisms of oxidative stress [8]. Therefore, GSH/GSSG and Cys/CySS couples in blood plasma represent a clinical measure of oxidative stress [4].

Here, it is assumed that individual's state of training could be important to determine the extent of redox homeostasis following acute bout of exercise [16–18], more notably the level of GSH [19]. Our hypothesis was that individuals with different history of physical activity should have different redox state and react in a different way encountering acute stress. To our knowledge, the changes in GSH redox status during exercise have been investigated in two human studies [16, 20]. Thus, the present study was designed to compare GSH redox status of individuals with three distinct exercise training statuses at rest and also in response to acute exercise.

## 2. Materials and Methods

### 2.1. Subjects

Thirty voluntary well-trained (*n* = 10, WT group), moderately trained (*n* = 10, MT group), and untrained (*n* = 10, UT group) male subjects participated in this study. Subjects from WT group were selected from elite soccer players who played in teams of the highest division of league in Ardebil. Based on self-reported frequency of physical training, the other groups were identified: moderately trained subjects with regular physical training (walking, jogging, bicycling, basketball, and soccer). Untrained subjects had no physical training or sport in their routine (more detailed information about subjects is seen in Table 1).

At the beginning of the experiment, the study protocol was approved by the Ethical Committee of Ardebil University, and then participants completed medical history questionnaire and signed informed consent. None of the participants showed signs of bacterial or viral infection symptoms. In addition, other exclusion criteria were drinking alcohol, smoking, and taking anti-inflammatory drugs or antioxidant supplements.

### 2.2. Procedures

Generally, this study was designed in two parts: the preliminary and main exercise trials.

#### 2.2.1. The Preliminary Trial

Two weeks prior to enrollment into the study, all subjects passed a physical examination and a maximal oxygen consumption (Vo_2_max) test.

#### 2.2.2. Subjects Characteristics

The subjects' weight and height were recorded using electronic scale (model 712; Seca, Germany) and portable Stadiometer (Holtain, UK), respectively, and then participants completed a body composition assessment.

#### 2.2.3. Maximal Oxygen Consumption (Vo_2_max)

All subjects performed an incremental test on a treadmill (Model 6150E, Sport Art, UK) using Bruce test [21] 1-2 h after breakfast.

#### 2.2.4. Dietary Assessment

All subjects kept their normal diet during the study period and completed daily food records until the day of experiment. Diet records were analyzed for total kilocalories, protein, carbohydrate, fat, vitamin C, vitamin E, vitamin A, antioxidants sources, and selenium intake using commercial software (Food Processor IV; Nutrition System, Iran).

#### 2.2.5. Exercise Protocol

Subjects completed exercise protocol including 5 min running with 50% Vo_2_max and 30 min running with 75% Vo_2_max on treadmill, while heart rate was continuously monitored using short-range telemetry (Polar S610, Polar Electro, Finland). Water consumption was encouraged throughout the main trial. Blood samples were taken before the exercise (following overnight fasting) and immediately, 10 min, and 30 min after the exercise protocol from an antecubital vein.

### 2.3. Outcome Measures

#### 2.3.1. Sample Preparation

The blood samples were transferred to four aliquots: the first vials containing EDTA were left at room temperature for 2 hours, and then they were used for measuring hemoglobin and hematocrit using automated Coulter Counter (Sysmex k-x21) in order to correct plasma volume shifts [22]. The second vials were centrifuged at 1600 g for 5 min for cortisol and creatine kinase (CK) measurement in serum, and the last vials containing EDTA were immediately transferred on ice and then centrifuged at 1600 g for 5 min. Erythrocytes were washed twice with cold 9% NaCl solution and were lysed by freezing for 2 hours. Finally, hemolysate (100 mL) was deproteinized by adding 400 mL of 6% MPA and 100 mL of glutathione ethyl ester as internal standard. Precipitated proteins were removed by centrifugation (7 min, 10 000 g, and 48°C). The resulting supernatant was used for measurement of GSH and total GSH in RBCs [23]. The fourth vials (1350 *μ*L) were centrifuged and were stored at −80°C until GSH/GSSG and Cys/CySS analysis. Another microtube labeled N containing 1350 *μ*L was centrifuged for 1 min in isolate RBC. Then, 200 *μ*L of supernatant was transported to a microcentrifuge tube labeled S and was stored at −80°C. This method of sample preparation and storage reduces probability of artifacts production during hemolysis or GSH thiol-disulfide exchange [5].

#### 2.3.2. Measurement of Serum Creatine Kinase Activity and Cortisol

Serum cortisol level was measured using chemiluminescent immunoassay and a commercial kit (Liaison, USA). Creatine kinase activity was measured by spectrophotometry using a commercial kit (Pars Azmoon Lab, Iran).

#### 2.3.3. Measurement of the Thiol and Disulfide Forms of Glutathione and Cysteine in Plasma and RBCs

High-performance liquid chromatography (HPLC) with fluorescence detection was used for plasma (with minor modifications) [5] and RBCs markers [23]. The HPLC analyses were performed with Agilent 1200 series HPLC systems equipped with a quaternary pump system (G1311A) and a fluorescence detector (G1321A) (Agilent Technologies, Waldbronn, Germany) by using reversed phase gradient elution on Eclipse XDB-C18 column (150, 4.6 mm; 5 *μ*m particle size). Briefly, samples in S tubes were spun for 2 min in microcentrifuge to precipitate protein. A 300 *μ*L aliquot of each supernatant (pH = 9.0 ± 0.2) was mixed with 300 *μ*L of the dansyl chloride solution for derivatization and placed in the dark at room temperature for 24 hours. Chloroform (500 *μ*L) was added to each tube to extract the unreacted dansyl chloride. An aliquot of the upper layer (20 *μ*L) was injected to the system. The mobile phase was composed of solvent A containing methanol/water (80/100) and solvent B was an acetate-buffered (pH = 4.6) methanol solution prepared by mixing 640 mL of methanol, 200 mL of acetate stock, 125 mL of glacial acetic acid, and 50 mL of water. The retention times of GSH, GSSG, Cys, and CySS were 19.1, 22.3, 10.1, and 8.4 min.

For any measurements in RBCs, first 100 *μ*L of precipitated proteins sample from hemolyzed erythrocytes was derivatized with 100 *μ*L of an ortho-phthalaldehyde solution and 800 *μ*L of 500 mM sodium phosphate (pH = 7.00). A mobile phase was composed of 50 mM of sodium acetate buffer (pH = 6.20) and acetonitrile. Fluorimetric detection was performed at 420 nm after excitation at 340 nm. The flow rate during elution was 0.7 mL/min, the retention time of GSH was 3.6 min, and the injection volume was 20 *μ*L. GSSG concentration was obtained from subtraction of the GSH from the total glutathione (GSHt) values assessed by this method performing a reduction step of GSSG with dithiothreitol before protein precipitation.

#### 2.3.4. Analysis

The results are presented as mean ± SEM, except for subject characteristics, which are presented as mean ± SD. All data were analyzed for their normal distribution using KS test. Subject characteristics, dietary data, and estimated percentage of changes in plasma volume were analyzed by using ANOVA. A univariate GLM for repeated measures was used to analyze the differences within groups and for fixed between-groups factors, Bonferroni test was used for multiple comparison tests. Also, ANOVA with Tukey post hoc test was used to analyze the differences between and within groups. Calculations were performed with the SPSS, Version 20.0 (SSPS Inc., Chicago, IL), statistical package. Statistical significance was defined as *p* < 0.05.

## 3. Results

The physiological characteristics of the participants are represented in Table 1. All subjects showed normal BMI with no significant differences between groups (WT group: 22.28 ± 1.87; MT group: 23.12 ± 3.01; UT group: 23.37 ± 2.74 kg/m^2^). The differences found in self-reported training questionnaire were confirmed by differences in aerobic fitness levels, as resulting from the maximal oxygen uptake (Vo_2_max) and body fat percentage. The well-trained group including 10 soccer players showed the highest training level (6.4 ± 0.33 h/week), MT group included 10 subjects with the intermediate training frequency (1.20 ± 0.16 h/week), and UT group included 10 subjects with the lowest Vo_2_max (43.63 ± 4.11 mL/min/kg) and greatest body fat percentage (15.98 ± 4.17%), with no physical training history in the recent 10 years. Neither of the groups showed significant changes in plasma volume during the experiment (Table 2). Also, no significant difference was observed in the calculated amount or composition of the 3-day food consumption before the main trial (Table 3).

### 3.1. Stress Hormone Response

Table 4 shows the mean (±SEM) values for serum cortisol concentration. It was increased significantly after exercise in the WT (*p* = 0.003), MT (*p* = 0.034), and UT (*p* < 0.001) groups compared to before exercise. There was no significant between-groups difference either in preexercise (*F* = 1.084, *p* = 0.365) or in postexercise (*F* = 0.536, *p* = 0.591) values.

### 3.2. Muscle Damage

Table 4 shows the mean (±SEM) values of serum CK activity. It was significantly increased immediately after exercise in all groups (*p* < 0.001). The greatest but insignificant preexercise value was observed in WT group compared with MT and UT group (*F* = 0.309, *p* = 0.737).

### 3.3. The Thiol Form to Disulfide Form Ratio of Cysteine (Cys/CySS) in Plasma

**Figure 1:**
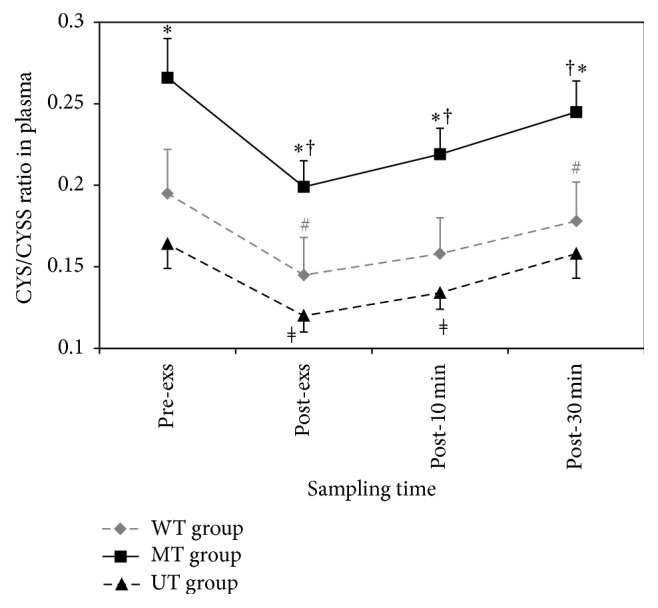
Effects of one session of aerobic exercise on plasma Cys/CySS in subjects with different physical training status. Values represent mean ± SEM (*n* = 10) for each time point. *∗* MT group versus WT and UT groups in pre-exs (*F* = 5.357, *p* = 0.011), post-exs (*F* = 5.600, *p* = 0.009), post-10 min (*F* = 6.656, *p* = 0.004), and post-30 min (*F* = 5.403, *p* = 0.011); # WT group by time (*p* < 0.001); † MT group by time (*p* = 0.001); *ǂ* UT group by time (*p* < 0.001).

As Figure 1 shows, the Cys/CySS ratio in MT group was significantly greater than in WT and UT groups in all of the recorded times: pre-exs (*F* = 5.357, *p* = 0.011), post-exs (*F* = 5.600, *p* = 0.009), post-10 min (*F* = 6.656, *p* = 0.004), and post-30 min (*F* = 5.403, *p* = 0.011). This ratio was significantly decreased immediately after exercise in all groups (*p* = 0.003) and after 10 min it was started to increase till 30 min after exercise. The decrease in the Cys/CySS ratio was secondary to the decrease in plasma Cys level and increase in CySS levels (Table 4).

### 3.4. The Redox State of GSH/GSSG in Plasma

**Figure 2:**
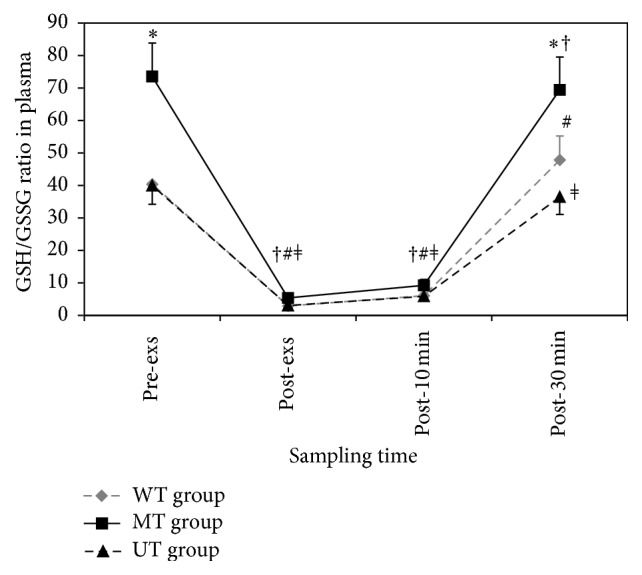
Effects of one session of aerobic exercise on plasma GSH/GSSG in subjects with different physical training status: values represent mean ± SEM. *∗* MT group versus WT and UT groups in basal (*F* = 6.666, *p* = 0.004) and end point (*F* = 4.536, *p* = 0.020) values. # (*p* < 0.001) WT group by time; † (*p* < 0.001) MT group by time; *ǂ* (*p* < 0.001) UT group by time.

Plasma level of GSH in MT group was significantly greater than in WT and UT groups in pre-exs (*F* = 5.853, *p* = 0.008), post-exs (*F* = 7.230, *p* = 0.003), post-10 min (*F* = 6.353, *p* = 0.005), and post-30 min (*F* = 5.026, *p* = 0.014). There was no significant change in plasma concentration of GSH following exercise in neither group (Table 4). Plasma GSH/GSSG showed a sharp significant reduction immediately after exercise (*p* < 0.001 in all groups) till the end of 10 min after exercise (WT: *p* = 0.001, MT and UT: *p* < 0.001) and then turned to strong rising till 30 min after the exercise in all groups (Figure 2). The preexercise value in MT group was significantly greater than in WT and UT groups at basal (*F* = 6.666, *p* = 0.004) and end point (*F* = 4.536, *p* = 0.020) values (Figure 2).

### 3.5. The Redox State of GSH/GSSG in Red Blood Cell (RBC)

Red blood cells GSH level was increased in MT (*p* = 0.012) and WT (*p* = 0.018) groups immediately after exercise and then started to reduce during the further time course of study in MT group. Immediately after exercise level of GSH in RBCs in MT group was significantly greater than in UT group, (*p* = 0.022, Table 4). Inversely, the untrained group showed significant reduction in GSH (*p* = 0.002). The level of GSSG in RBCs in MT group was lower than in WT group immediately (*p* = 0.008) and 10 min (*p* = 0.025) after exercise. Interestingly, GSSG level in untrained (*p* = 0.004) and well-trained individuals showed a significant increase (*p* = 0.006) immediately after exercise (Table 4).

**Figure 3:**
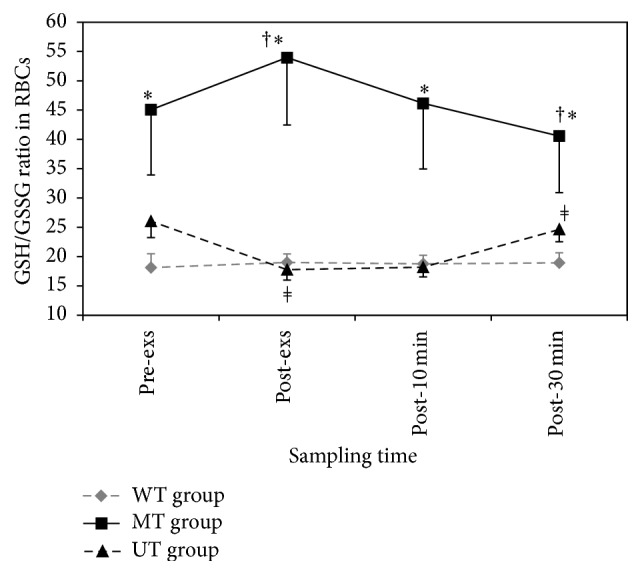
Effects of one session of aerobic exercise on GSH/GSSG ratio in red blood cell (RBC) in subjects with different physical training status: values represent mean ± SEM (*n* = 10) for each time point. *∗* (*F* = 4.176, *p* = 0.026) MT group versus WT and UT groups in pre- and post-exs (*F* = 9.202, *p* = 0.001), post-10 min (*F* = 5.890, *p* = 0.008), and post-30 min (*F* = 3.737, *p* = 0.037); † MT group by time (*p* < 0.001); *ǂ* UT group by time (*p* = 0.006).

The changes of GSH/GSSG in RBCs are shown in Figure 3. Immediately after exercise, there was a statistically significant decrease in UT group (*p* = 0.003) and there was a statistically significant increase in MT group (*p* = 0.007), whereas the well-trained subjects did not show any significant changes (*p* = 1.000). The ratio of GSH/GSSG in RBCs in MT group was significantly greater than in WT and UT groups for preexercise (*F* = 4.176, *p* = 0.026), post-exs (*F* =9.202, *p* = 0.001), post-10 min (*F* = 5.890, *p* = 0.008), and post-30 min (*F* = 3.737, *p* = 0.037).

## 4. Discussion

The aim of this study was to evaluate the changes in glutathione redox ratio expressed as GSH/GSSG and Cys/CySS in plasma and also GSH/GSSG in RBCs in subjects with different physical training status. Our results showed that physical training status affected the plasma GSH/GSSG and Cys/CySS ratio and RBCs GSH/GSSG ratio at baseline and after exercise.

All groups experienced one session of physical stress and showed cortisol elevation without significant between-groups differences, excluding the possibility of hypothalamus-adrenal axis adaptation in WT group [24].

Also, this finding confirms that exercise with 75% Vo_2_max can be a physiological stress for all subjects independently of their physical fitness status. This finding is in agreement with other previous studies [25–27].

No significant difference in preexercise values of CK reflects that groups were well matched in terms of previous muscle damage and inflammation. Following exercise, CK showed elevation in all groups with no between-groups differences. Elevation in serum creatine kinase in all groups probably reflects exercise-induced muscle damage in sarcomeres Z disk [28] or change in permeability of the muscle vasculature [29, 30]. Our findings are in agreement with studies showing an increase in CK following high intensive aerobic exercise in untrained, moderately trained [31], and well-trained [32] individuals.

Preexercise analysis revealed the highest level of plasma GSH and GSH/GSSG in MT group with moderate Vo_2_max (52.76 ± 2.62 mL/min/kg) and training frequency (1.20 ± 0.16 h/week). On the other hand, WT group with more Vo_2_max (60.90 ± 3.96 mL/min/kg) and also training frequency (6.4 ± 0.33 h/week) showed the lowest plasma levels of GSH/GSSG. The lowest GSH/GSSG might be related to chronic oxidative stress and poor antioxidant capacity resulting from previous strenuous training in well-trained athletes [33]. The novelty of this study compared to similar investigations [20, 34–36] is that here we considered exercise effect on oxidative stress and cellular redox state in three distinct physical training statuses of well-trained, moderately trained, and untrained subjects. To our knowledge, no study considered the effect of physical activity with moderate intensity on redox state, and there are discrepancies in obtained data. For example, Pittaluga et al. in their study reported positive relationship between GSH/GSSG and Vo_2_max in athletes [36], and Michelet et al., similar to what we did, reported the highest GSH/GSSG in individuals with habitual physical training [37].

As mentioned before, glutathione couple GSH/GSSG is a critically important redox biomarker [38] which together with Cys/CySS participates in homeostasis of cellular redox [39]. For example, GSH/GSSG is involved in storage and transport of nitric oxide, reducing ribonucleotides to deoxyribonucleotides, processing of some proteins, interfering in redox signaling pathways, detoxification of xenobiotics [40], and finally protecting cells from oxidative stress [12]. Our data suggest that subjects from MT group with higher GSH/GSSG ratio are predicted to be healthier than those from WT group. Probable reasons could be more antioxidant enzymes in MT or more cells damage due to chronic oxidative stress in WT group [41]. However, changes in antioxidant enzymes and oxidative stress markers remain to be elucidated in the future studies.

In addition, plasma analysis revealed a significant reduction in GSH/GSSG and Cys/CySS in all groups, reflecting exercise-induced oxidative stress. Considering the fact that GSH/GSSG and Cys/CySS couples in blood plasma stand for clinical measure of oxidative stress [4], reduction in plasma GSH/GSSG causes a transient shift in redox balance towards a more oxidizing environment. Since consumption of GSH or increase in GSSG production could shift redox balance towards a more oxidizing environment, the decrease in plasma GSH/GSSG might be related to increase in leakage of GSSG from the cells [40–43]. In other words, reduction in plasma GSH/GSSG is secondary to elevation in plasma GSSG levels [44–47]. However, consumption of GSH following intensive acute exercise due to elevation in ROS cannot be excluded too [11]. Similar to our finding, reduction in plasma GSH has been reported by Viguie et al. [44]. In order to ensure redox homeostasis, the secretion of GSH from the liver to the plasma [48] plays a pivotal role.

Regarding the fact that changes in plasma thiols, especially oxidized glutathione and GSH/GSSG ratio, have been used as markers of oxidative stress status in biological systems [4, 10, 42], significant increase in GSSG and further decrease in the GSH/GSSG immediately after the exercise probably indicate elevation in free-radical production [44, 49]. We should emphasize here that the changes in the GSSG levels and GSH/GSSG ratio were transient and returned to the baseline value 30 min after the exercise.

Finally, RBCs GSH/GSSG ratio showed no change, elevation, and reduction in WT, MT, and UT groups, respectively. The lowest level of RBC GSH/GSSG and the highest level of GSSG in WT group compared with other groups indicate the lowest reducing power in red blood cells in this group. The possible explanation for this finding might be that chronic production of free radicals may overwhelm the capacity of the antioxidant defense system and leads to a permanent shift in redox balance towards a more oxidizing environment [6] due to a prolonged increase in ROS levels, in accordance with the principle of hormesis [49]. In other words, the cells may enter a state of “chronic oxidative stress” that induces upregulation of the antioxidant potential with enhancement in metabolism and energy consumption to replace consumed GSH and/or transport it to the places where it is needed [12].

In moderately trained subjects, RBCs GSH/GSSG ratio was increased following exercise. This increase was secondary to increase in GSH and decrease in GSSG. The exact physiologic mechanisms of this increase have not been understood yet [50], but activation of MAPK and NF-*κ*B in the inflammation signaling pathways in an effort to restore redox balance could be one reason for elevation of GSH/GSSG. Thus, exercise-induced changes in the glutathione system seem to be effective in RBCs and may prevent ROS-induced cell damage [20, 51]. Therefore, these participants benefit from sufficient antioxidant power based on hormetic-associated upregulation of antioxidant defense [7, 20, 52].

In untrained young men (UT group), the GSH level and GSH/GSSG ratio in RBCs decreased and GSSG level increased following exercise. Thus, these participants are predicted to suffer from inadequate level of RBCs antioxidant protection system encountering exercise and are predisposed to RBC damage and further related diseases [53].

It is important to notice that selected body fluid is important in detection of redox biomarkers [38]. Glutathione and its disulfide form are found in all extracellular biological fluids, including plasma, interstitial fluid, cerebrospinal fluid, alveolar lining fluid, saliva, bile, pancreatic fluid, tears, sweat, and urine [54]. Here, we chose blood as an extracellular fluid circulating between body cells and exchanging redox biomarkers according to the metabolism and physiological states [38]. In addition, blood plasma and cells are a noticeable generator of reactive species. In fact, blood plasma contains metal ions and oxidized metabolites (glucose, albumin, and fatty acids) which potentially could generate reactive species [55].

It is important to notice that distribution of GSH and GSSG among body fluids and tissues is not equal. For example, in our study, the concentration of GSH in RBCs of UT and WT groups was 1400- and 12300-fold higher than plasma, which confirms the previous report about fluid and tissue GSH relationship [54, 56]. Finally, considering the finding that the changes in RBC GSH/GSSG are not parallel with plasma GSH/GSSG suggests that plasma redox biomarkers might not accurately reflect tissue redox status compared with RBC biomarkers. As mentioned above, one limitation of the present work is lack of data on cell oxidative stress biomarkers after an acute exercise which is suggested for future studies.

## 5. Conclusion

Our results point to the conclusion that the effect of high intensity acute exercise on glutathione redox ratio depends on physical training status of individuals. Therefore, it seems that a lifestyle with moderate regular exercise training will improve health by shifting in “redox” balance towards more reducing environment, encountering stressful conditions.

## Figures and Tables

**Table 1:** Subjects' characteristics of WT, MT, and UT groups.

Characteristics	WT group	MT group	UT group	*F*	*p* value
Age (yr)	21.10 ± 1.72	21.70 ± 1.88	20.10 ± 1.44	2.264	0.123
Weight (kg)	69.00 ± 6.94	69.40 ± 9.81	73.20 ± 9.47	0.688	0.511
Height (cm)	176.00 ± 7.87	173.20 ± 5.78	176.90 ± 4.28	0.981	0.388
BMI (kg/m^2^)	22.28 ± 1.87	23.12 ± 3.01	23.37 ± 2.74	0.485	0.621
Vo_2_max (mL/kg/min)	60.90 ± 3.96	52.76 ± 2.62	43.63 ± 4.11	56.538	<0.001^*∗*^
Body fat %	9.15 ± 0.96	11.68 ± 1.74	15.98 ± 4.17	16.669	<0.001^*∗*^
Years of training (yrs)	≈10.00	≈10.00	0	—	—
Training (h·week^−1^)	6.4 ± .33	1.20 ± 0.16	0	—	—

Data are mean ± SD. ^*∗*^A significant between-groups difference tested by ANOVA with Tukey post hoc test.

**Table 2:** Estimated percentage of changes in plasma volume in the groups of WT, MT, and UT.

Parameter	Pre-exs	Post-exs	Post-10 min	Post-30 min	*F*	*p* value
Plasma volume change, WT group	—	−0.71 ± 1.80	2.63 ± 1.47	2.78 ± 1.55	0.087	0.917
MT group	—	−0.78 ± 0.53	1.70 ± 1.30	1.41 ± 1.47	1.242	0.305
UT group	—	−0.49 ± 1.22	0.85 ± 1.97	−0.68 ± 2.28	0.939	0.403

Data are mean ± SEM. *p* value calculated using ANOVA with Tukey post hoc test.

**Table 3:** Dietary intake assessment during the 3-day period prior to the main trial.

	WT group	MT group	UT group	*F*	*p* value
Kilocalories	2817.40 ± 220.66	2864.70 ± 156.16	2455.20 ± 110.29	1.766	0.190
Protein	106.11 ± 10.17	106.27 ± 6.35	96.34 ± 5.46	0.558	0.579
Carbohydrate	466.65 ± 47.43	421.49 ± 33.24	348.58 ± 31.41	2.452	0.105
Fat total	59.49 ± 3.44	77.61 ± 2.60	70.75 ± 8.50	2.760	0.081
SFA	18.79 ± 2.45	23.35 ± 2.65	17.19 ± 2.99	1.388	0.267
MUFA	16.14 ± 2.82	24.72 ± 2.20	18.71 ± 2.77	2.829	0.077
PUFA	14.26 ± 2.27	20.10 ± 2.61	21.12 ± 4.10	1.423	0.258
Vitamin C	39.48 ± 9.56	42.54 ± 10.61	57.32 ± 20.85	0.427	0.657
Vitamin E	15.91 ± 2.96	24.62 ± 2.68	18.82 ± 2.53	2.630	0.090
Vitamin A total	251.41 ± 34.32	213.55 ± 29.45	153.48 ± 30.66	2.450	0.105
Carotene	79.40 ± 9.01	74.30 ± 9.16	51.70 ± 9.79	2.496	0.101
Selenium	0.05 ± 0.01	0.08 ± 0.02	0.06 ± 0.01	0.593	0.560

Gram quantities for each macronutrient are provided. Vitamin C and vitamin E are provided in milligrams. Vitamin A values are provided in retinol equivalents. Data are mean ± SEM. *p* value calculated using ANOVA.

**Table 4:** Biochemical parameters.

	Time	WT group (mean ± SEM)				MT group (mean ± SEM)				UT group (mean ± SEM)			
		Pre	Post	10	30	Pre	Post	10	30	Pre	Post	10	30
Plasma	Cys	9.90 ± 0.80	8.65 ± 0.91	9.12 ± 0.86	9.68 ± 0.86	13.79 ± 0.69^*∗*^	11.87 ± 0.69^*∗*^	12.65 ± 0.63^*∗*^	13.48 ± 0.68^*∗*^	12.11 ± 0.35	10.88 ± 0.45	11.32 ± 0.33	12.03 ± 0.34
	CySS	56.04 ± 5.82	65.94 ± 6.72	63.02 ± 6.44	58.85 ± 5.75	56.03 ± 6.25	62.71 ± 6.11	60.96 ± 5.93	58.21 ± 5.86	78.56 ± 6.37^#^	96.61 ± 8.85^#^	89.23 ± 7.40^#^	82.04 ± 7.12^#^
	GSH	1.98 ± 0.34	1.60 ± 0.25	1.73 ± 0.27	1.93 ± 0.36	3.22 ± 0.29^¶^	3.01 ± 0.38^¶^	2.65 ± 0.20^¶^	3.02 ± 0.28^¶^	1.80 ± 0.31	1.56 ± 0.26	1.57 ± 0.20	1.71 ± 0.27
	GSSG	0.04 ± 0.00	0.59 ± 0.09	0.31 ± 0.05	0.04 ± 0.00	0.05 ± 0.00	0.66 ± 0.10	0.36 ± 0.05	0.05 ± 0.00	0.050 ± 0.00	0.63 ± 0.10	0.34 ± 0.05	0.05 ± 0.00
RBCs	GSH	2.11 ± 0.25	2.87 ± 0.23	2.57 ± 0.16	2.34 ± 0.14	2.29 ± 0.13	3.01 ± 0.16^¥^	2.65 ± 0.15	2.22 ± 0.15	2.52 ± 0.16	2.24 ± 0.15	2.33 ± 0.18	2.49 ± 0.17
	GSSG	0.12 ± .01	0.15 ± 0.01	0.14 ± 0.01	0.13 ± 0.01	0.08 ± 0.01	0.08 ± 0.01^Δ^	0.08 ± 0.01^Δ^	0.08 ± 0.01	0.10 ± 0.01	0.13 ± 0.01	0.13 ± 0.1	0.10 ± 0.01
Cortisol		10.11 ± 1.38	14.24 ± 1.24	—	—	12.65 ± 1.36	16.08 ± 1.54	—	—	11.78 ± 0.98	14.77 ± 1.05	—	—
Creatin kinase		167.20 ± 15.25	185.20 ± 15.25	—	—	150.20 ± 17.62	172.50 ± 20.23	—	—	148.00 ± 24.02	167.4 ± 23.42	—	—

The superscript symbols indicate a significant between-groups difference tested by ANOVA with Tukey post hoc test and a significant within-groups difference tested by ANOVA for repeated measure with the following *p* value. *Plasma Cys levels*: ^*∗*^MT group versus WT group pre-exs (*p* = 0.001), post-exs (*p* = 0.010), post-10 min (*p* = 0.002), and post-30 min (*p* = 0.001); WT group by time (*p* < 0.001); MT group by time (*p* < 0.001); and UT group by time (*p* = 0.005); *plasma CySS levels*: ^#^UT group versus WT and MT groups in pre-exs (*F* = 4.464, *p* = 0.021), post-exs (*F* = 6.517, *p* = 0.005), post-10 min (*F* = 5.657, *p* = 0.009), and post-30 min (*F* = 4.675, *p* = 0.018); WT group by time (*p* < 0.001); MT group by time (*p* < 0.001); and UT group by time (*p* < 0.001); *plasma GSH levels*: ^¶^MT group versus WT and UT groups in pre-exs (*F* = 5.853, *p* = 0.008), post-exs (*F* = 7.230, *p* = 0.003), post-10 min (*F* = 6.353, *p* = 0.005), and post-30 min (*F* = 5.026, *p* = 0.014); *plasma GSSG levels*: WT group by time (*p* < 0.001), MT group by time (*p* < 0.001), and UT group by time (*p* < 0.001); *RBCs  GSH levels*: ^¥^MT group versus UT group at post-exs (*p* = 0.022), WT group by time (*p* = 0.017), MT group by time (*p* < 0.001), and UT group by time (*p* = 0.001); *RBCs GSSG levels*: ^Δ^MT group versus WT group at post-exs (*p* = 0.008) and post-10 min (*p* = 0.025), WT group by time (*p* < 0.001), and UT group by time (*p* = 0.001). The values of *cortisol* and *creatin  kinase* were increased after exercise compared to pre-exs (*p* < 0.05; tested with paired *t*-test).
